# Production and Characterization of Poly(lactic acid) and Poly(ε-caprolactone) Films Enriched with Pomegranate Peel Extract: Toward Biodegradable and Sustainable Food Packaging

**DOI:** 10.3390/polym18070896

**Published:** 2026-04-07

**Authors:** Ömer Faruk Uslu, Nebahat Aral, Sinem Argün, Özge Taştan Ülkü

**Affiliations:** 1 Department of Materials Science and Nanotechnology Engineering, Faculty of Engineering and Natural Sciences, Yeditepe University, Ataşehir, 34755 İstanbul, Türkiye; omerfaruk.uslu@std.yeditepe.edu.tr (Ö.F.U.); nebahat.aral@yeditepe.edu.tr (N.A.); 2 Department of Food Engineering, Faculty of Engineering and Natural Sciences, Yeditepe University, Ataşehir, 34755 İstanbul, Türkiye; sinem.argun@yeditepe.edu.tr; 3 Department of Biotechnology, Graduate School, Yeditepe University, Ataşehir, 34755 İstanbul, Türkiye

**Keywords:** poly(lactic acid) (PLA), poly(ε-caprolactone) (PCL), pomegranate peel extract (PoPE), packaging, biodegradable film, biodegradable composites, melt extrusion, film characterization

## Abstract

Recently, more sustainable and biodegradable packaging materials have begun to attract attention in food packaging due to major, rising concerns related to plastic usage. This study aims to develop and characterize biodegradable food packaging materials, namely poly(lactic acid) (PLA) and poly(ε-caprolactone) (PCL) enriched with pomegranate peel extract (PoPE). Firstly, the optimal extract selected was a 24 h maceration of PoPE with 60% ethanol, after production with different solvents and methods. PLA- and PCL-based films were produced via melt compounding with the addition of PoPE at different concentrations (1, 3, 5 and 10%, *w*/*w*). FTIR confirmed that the PoPE did not modify the chemical backbones of PLA or PCL, with only a more pronounced O–H band in PCL, suggesting mainly non-covalent/physical interactions. UV–Vis spectroscopy showed tunable warm coloration and strong UV shielding with reduced transparency; for PLA ~3–5 wt.%, PoPE enabled near-complete UV blocking, while PCL achieved very high UV protection even at low loadings. PoPE improved toughness in PLA (3–5 wt.%) and maintained ductility in PCL (1–10 wt.%). PoPE-added PLA and PCL films maintained thermal stability up to 10 wt.% according to TGA results. DSC/XRD indicated a matrix-dependent crystallization response. PLA remained largely amorphous, whereas PoPE promoted PCL crystallinity without changing polymer crystal polymorphs. SEM images revealed homogenous dispersion of PoPE in the films.

## 1. Introduction

The food packaging sector is under increasing pressure to reduce its environmental footprint while maintaining or improving the safety and quality of packaged products. Conventional plastics used in food packaging, such as PET and HDPE, offer excellent mechanical strength, processability, and barrier performance, but their persistence in the environment and dependence on finite fossil resources have raised major concerns related to plastic waste accumulation in nature [1]. In response, there has been growing interest in biodegradable polymers that can provide comparable performance in packaging applications while enabling more sustainable end-of-life scenarios [2]. At the same time, the use of conventional synthetic stabilizers and functional additives is being critically re-evaluated in food-contacting materials due to issues of migration, potential toxicity, and tightening regulatory constraints [3,4]. This has created a strong demand for packaging materials in which both the polymer matrix and the key additives originate, as much as possible, from biodegradable, food-compatible sources.

Among the different strategies proposed, biodegradable aliphatic polyesters have attracted particular attention for use in flexible and semi-rigid food packaging [2]. These materials can be processed with conventional extrusion and film-forming equipment, offer tunable mechanical and optical properties, and can be engineered to degrade under specific conditions. However, ensuring the stability of such polymers during processing and throughout the shelf life of the packaged product remains a critical challenge. Food packaging is routinely exposed to elevated temperatures, oxygen, moisture, and light, all of which can promote polymer degradation and loss of performance [4]. To design viable bio-based packaging, it is therefore essential not only to select suitable polymer matrices but also to identify natural additives that can protect the material, shield the food from light-induced deterioration, and contribute to overall formulation sustainability.

Poly(lactic acid) (PLA) and poly(ε-caprolactone) (PCL) are two key biodegradable polyesters widely considered for packaging applications [2,5]. PLA, produced from renewable feedstocks such as corn or sugarcane, offers high stiffness and strength, with good transparency and gloss, and is industrially compostable, but it is relatively brittle, has limited thermal resistance (glass transition temperature (T_g_) ≈ 55–65 °C), and is susceptible to thermal, thermo-oxidative, and photo-oxidative degradation, which can cause chain scission, yellowing, and loss of properties [2]. In contrast, PCL is currently mostly fossil-based but fully aliphatic and biodegradable, with a lower melting point (≈60 °C), higher chain flexibility, and typically higher crystallinity, leading to soft, highly ductile films suitable for flexible packaging where toughness is required. PCL also shows good compatibility with hydrophobic bioactive molecules, which facilitates the incorporation of natural additives, yet it is sensitive to thermal and thermo-oxidative degradation and to structural changes under prolonged heat and UV exposure [6]. Understanding how stabilizing additives interact with these two matrices, and how their different chemistry and morphology affect the balance between flexibility, stability, and processability, is therefore crucial for designing robust, biodegradable packaging formulations.

Plant extracts, which are rich in polyphenols and flavonoids, function as natural antioxidants and antimicrobials. These compounds can influence polymer crystallinity, thermal transitions, and surface morphology [7,8]. The interaction between polymer matrices and plant-based additives changes film properties, thus requiring combined testing to improve performance [9,10]. Recent work has shown that fruit-derived extracts can act as natural antioxidants and functional additives in biodegradable polyester-based active packaging. For example, PLA films incorporating orange peel extract or other fruit byproduct extracts have been developed as active packaging materials, improving oxidative stability and contributing to more sustainable formulations [11,12]. Similarly, electrospun PCL nanofibers loaded with grapefruit seed extract have been proposed for active food packaging, where the polyphenol-rich extract enhances antioxidant performance and helps protect the packaged product [13]. In this context, pomegranate peel is another abundant byproduct of the pomegranate juice and food-processing industries. Pomegranate peel extract (PoPE) is rich in polyphenolic compounds such as punicalagin, ellagic acid, and other flavonoids. Also, it exhibits strong radical-scavenging activity and pronounced UV absorption [14,15]. These features make PoPE a promising multifunctional additive for polymer-based food packaging films. At appropriate loadings, it can act as an internal antioxidant that delays thermo-oxidative degradation during melt processing and storage, and as a UV screen that reduces the transmission of harmful UV radiation to the packaged food. This combination is particularly valuable where oxidation and light exposure drive color, flavor and nutritional changes, and the valorization of pomegranate peel as an additive source also supports circular-economy strategies by converting a low-value residue into a functional component of advanced packaging materials. In a recent study, combined incorporation of pomegranate peel powder (PoP) and PoPE enhanced the physical properties of sweet potato starch-based films, especially by improving tensile strength, water solubility and barrier properties [16]. Furthermore, the addition of PoPE into chitosan films influenced physical properties by increasing moisture content and water vapor permeability, while there were no notable changes in optical properties and film thickness. The incorporation of lower extract concentrations enhanced thermal stability; however, higher concentrations improved functional properties rather than strengthening the physical properties of the films [17].

While previous research has investigated the mechanical and thermal properties of PLA and PCL films that contain various extracts [18,19,20], studies specifically addressing PoPE-containing systems have mainly focused on PLA-based matrices. For example, Andrade et al. developed active PLA films containing PoPE, Dai et al. reported on PLA/ZnO/PoPE films, and Bodbodak et al. prepared PLA/HPMC nanofibers loaded with PoPE [21,22,23]. By contrast, closely related PCL-based PoPE matrices are rare and have been reported mainly in electrospun biomedical systems rather than packaging films, such as the PCL/PoPE nanofibrous wound dressing reported by Wang et al. [24]. Although pomegranate-derived additives have already been explored in PLA and multicomponent matrices, a direct comparison of the same extract in both PLA and PCL matrices under comparable conditions is still lacking.

In short, the objective of this study is to produce and characterize PLA and PCL films enriched with pomegranate peel extract (PoPE) as biodegradable and functional packaging materials. The goal of examining these matrices separately is to understand how the same bioactive additive interacts with and alters the properties of a hard, glassy polymer (PLA) versus a flexible, rubbery polymer (PCL). The study focuses on determining the morphological alterations, thermal stability, natural coloring effects, UV-barrier efficiency, and mechanical modifications caused by the extract in each polymer matrix.

## 2. Materials and Methods

### 2.1. Materials

PLA Luminy^®^ LX175 was purchased from Total Corbion (Rayong, Thailand), and Poly(ε-caprolactone) (average Mn 80,000) was purchased from Sigma-Aldrich (St. Louis, MO, USA). According to the supplier’s product data sheet, Luminy^®^ LX175 is a high-viscosity, low-flow, amorphous, transparent PLA resin with a reported stereochemical purity of 96% (L-isomer). Pomegranate peels were supplied from DOHLER (İzmir, Türkiye) in powder form. DPPH (2,2-Diphenyl-1-picrylhydrazyl), ABTS (2,2-azino-bis(3-ethylbenzothiazoline-6-sulfonic acid)), potassium persulfate, Trolox (6-hydroxy-2,5,7,8-tetrame thylchroman-2-carboxylic acid), gallic acid, sodium carbonate and hydrochloric acid were purchased from Sigma-Aldrich. Ethanol (99.9%), methanol (99.9%), and Folin–Ciocalteu reagent (2 N) were obtained from Merck (Rahway, NJ, USA).

### 2.2. Methods

#### 2.2.1. Production of PoPE

The extraction of phenolic-antioxidant compounds from dried pomegranate peel waste (PoP) was carried out using maceration and ultrasound-assisted extraction methods. The maceration was performed using PoP prepared with a 1:30 (*w*/*v*) PoP-to-solvent ratio at 100 rpm and 40 °C for 1 h and 24 h in a shaking incubator (New Brunswick, INNOVA 40, Dieppe, Canada). Distilled water (DW), 60% methanol (M), and 60% ethanol (E) were used as extraction solvents. Ultrasound-assisted extraction (US) was carried out using an ultrasonic water bath (Bandelin, DT 510 H, 35 kHz, Berlin, Germany) at 40 °C for 10, 20, and 30 min with the same PoP-to-solvent ratio. Distilled water and an ethanol–acid–water mixture (EA) (60:5:35, (*v*/*v*/*v*)) were used as extraction solvents. Following extraction, the mixture was filtered through filter paper and then passed through a 0.45 µm filter. The solvent in the extract was evaporated using a rotary evaporator (Buchi, Rotovapor RII, Flawil, Switzerland) at 40 °C until the extract reached 35 ± 0.5 °Bx (degrees Brix), which indicates the total soluble solids content in the extract. The obtained extract was in a viscous form with a moisture content of 60–65%. The PoPE were stored at 4 °C until analysis.

#### 2.2.2. Characterization of PoPE

##### Extraction Yield

Extraction yield was determined by evaporating the solvent at the end of the extraction, drying the remaining extract, and weighing it. First, PoP and a glass flask were weighed using an analytical balance and the extraction solution was prepared with a 1:30 (*w*/*v*) PoP-to-solvent ratio. Then, the solvent in the extract was evaporated at 40 °C using a vacuum rotary evaporator. The extract in the glass flask was freeze-dried and weighed. Extraction yield was calculated using the following formula:

Extraction yield = (Weight of extract after drying)/(Weight of PoP) × 100(1)

##### Determination of Total Phenolic Content (TPC) of PoPE

The total phenolic content (TPC) of PoPE was determined according to Çelebi et al. [25]. The extract was diluted to 1:100 before analysis. First, 200 µL of the diluted extract was mixed with 1 mL of 0.2 N Folin–Ciocalteu reagent. Then, 800 µL of 7.5% Na_2_CO_3_ was added to the mixture. Absorbance values were measured at 765 nm using a microplate reader spectrophotometer (Thermo Scientific, MultiSkan Go RE 4.1) after 1 h of incubation at 25 °C. TPC was expressed as mg gallic acid equivalent/g (mg GAE/g dry sample).

##### Determination of Antioxidant Activity of PoPE

The antioxidant activity of PoPE was determined using DPPH (2,2-diphenyl-1-picrylhydrazyl) and ABTS^•+^ radical scavenging activity methods. For the calibration curve, 0.1 mM Trolox solution was diluted with methanol to obtain concentrations of 0.05, 0.10, 0.25, 0.50, 0.75, and 1.00 mM, and their absorbance values were measured at 517 nm using a microplate reader.

The antioxidant activity of PoPE was determined using the DPPH method according to Çelebi et al. [25]. First, the extracts were diluted 1:100 with 60% ethanol. A 0.1 mM DPPH solution was prepared using methanol. Then, 0.1 mL of the extract was mixed with 2 mL of 0.1 mM DPPH solution. The mixture was incubated in the dark at room temperature for 30 min. Afterward, the absorbance of the sample was measured at 517 nm using a microplate reader UV-VIS spectrophotometer. Results were expressed as ‘mM Trolox/g’. Additionally, DPPH radical scavenging activity (%) was calculated as follows.

DPPH Radical Activity (%) = [(*A*_0_ − *A*_s_)/*A*_0_)] × 100 (2)

In the equation, ‘A_s_’ represents the absorbance of the extract, and ‘A_0_’ represents the absorbance of the blank sample.

The antioxidant activity of PoPE was determined using the ABTS^•+^ radical scavenging activity [25]. Before analysis, ABTS (2,2¢-Azino-bis-(3-ethyl-benzthiazoline- sulphonic acid)) reagent was diluted with distilled water until the absorbance at 734 nm was 0.70 ± 0.02. First, 20 μL of PoPE (diluted 1:200) was mixed with 200 μL of ABTS^•+^ solution and left in the dark at room temperature for 30 min. The absorbance of the samples was measured at 734 nm using a microplate reader. Results were expressed as ‘mM Trolox/g sample’. Also, ABTS^•+^ radical activity (%) was calculated as follows:

ABTS^•+^ Radical Activity (%) = [(*A*_0_ − *A*_s_)/*A*_0_)] × 100 (3)

‘A_s_’ in the equation represents the absorbance of the pomegranate peel extract, and ‘A_0_’ represents the absorbance of the blank sample.

#### 2.2.3. Preparation of Films

##### Compounding

To prepare the PoPE-containing films, pelletized PLA and PCL were melt compounded with PoPE in the form of a viscous extract (35°Bx) in a RTX-M40 internal mixer (Kırklareli, Türkiye). Processing temperatures were selected according to the melting behavior of each polymer, while the rotor speed, mixing time, and batch size were kept constant for a better comparison between formulations. For film preparation, the optimum extract used was a 24 h maceration with 60% ethanol.

The PLA and PCL pellets were dried at 50 °C for 6 h before processing. The PoPE and PCL were compounded in an internal mixer at 130 °C, 10 rpm for 5 min, and the PoPE and PLA were compounded in an internal mixer at 195 °C, 10 rpm for 5 min to achieve a uniform blend. Each batch was prepared with a total mass of 40 g, adjusting the PoPE loading to 0, 1, 3, 5, and 10 wt.%. The resulting samples are listed in Table 1.

An internal mixer was selected as a laboratory-scale formulation-screening method because it enables controlled comparison with small material quantities. However, compared with twin-screw extrusion, it provides different shear and residence-time histories, which may affect dispersion and morphology; therefore, absolute values may differ under continuous extrusion.

##### Film Formation

After mixing, the resulting composites were converted into films by hot-pressing at a pressure of under 2 bar for 4 min. The PLA films were pressed at 190 °C, and the PCL films were pressed at 75 °C. Each film was around 8 g. The pressed films were taken out of the press and allowed to cool down at room temperature. Since film formation was performed by hot pressing without a controlled film-drawing/stretching step, the control of the final thicknesses of PLA and PCL films produced under the same conditions remained limited, and the average thickness values of the films are given in Table 1.

#### 2.2.4. Characterization of Films

##### Fourier-Transform Infrared (FTIR) Spectroscopy

PLA and PCL films and PoPE at different temperatures were subjected to FTIR analysis using a Thermo Fisher Scientific Inc. Nicolet IS50 ATR (Waltham, MA, USA). Every sample was examined at a frequency range between 400 and 4000 cm^−1^. Each sample was measured in three separate locations to ensure the reliability of the results. Also, the PoPE samples were heated in an oven for 10 min at 130 and 190 °C to examine the effect of temperature on the PoPE.

##### UV–Vis Spectroscopy

UV–Vis spectroscopy measurements were carried out using a NKD 7000 model (Aquila, Saffron Walden, UK) in the 280–1000 nm wavelength range. Film strips were measured in transmission mode at room temperature, and three measurements were collected at different spots for each sample. Transmittance spectra were recorded against air as a reference to determine the light transmission and UV-blocking performance of the films in the UV and visible regions.

Because optical transmittance depends on optical path length, thickness differences among films can contribute to their observed transparency and UV-shielding behavior in addition to composition-related effects. Accordingly, absolute transmittance values should be interpreted with caution when comparing samples with different thicknesses, particularly across PLA and PCL matrices. In this study, the optical trends are therefore emphasized mainly within each polymer series and in relation to PoPE loading.

##### Color Analysis

Color parameters of the samples were determined using a colorimeter (Konica Minolta CM-5, Osaka, Japan). For each specimen, three measurements were taken and averaged to obtain L* (lightness), a* (red/green coordinate), and b* (yellow/blue coordinate). Chroma (C*) and hue angle (°Hue) were subsequently calculated according to Equations (1), (2), and (3), respectively. The overall color difference (ΔE*) relative to the PLA ref and PCL ref film was also computed, and ΔE values greater than 3 were regarded as visually noticeable to the human eye [26].

(4) $${\Delta E}^{*}=\sqrt{({{\Delta L}^{*})}^{2}+({{\Delta a}^{*})}^{2}+({{\Delta b}^{*})}^{2}}$$

(5) $$C^{*}=\sqrt{{{(a}^{*})}^{2}+{{(b}^{*})}^{2}}$$

(6) $${}^{\circ}Hue={tan}^{-1}\left(\frac{b}{a}\right)$$

##### Tensile Test

The tensile properties of the PLA-based films were measured according to ASTM D882 [27] using an Instron 5966 (Norwood, MA, USA). Rectangular strips with a width of 20 mm and an initial gauge length of 50 mm were tested at a crosshead speed of 10 mm/min at room temperature. A similar ASTM D882-based approach for characterizing PLA film tensile behavior has also been reported in previous studies on PLA-based packaging films [28].

For the PCL-based samples, preliminary tests were also carried out on film strips following ASTM D882. However, due to the very high ductility of the PCL matrix, the specimens reached the maximum crosshead travel of the testing machine without failing, and a true elongation at break could not be determined. To obtain reliable fracture data, PCL samples were therefore tested using dog-bone specimens prepared in accordance with ASTM D638 [29]. Type IV specimens with an initial gauge length of 25 mm were tested at a crosshead speed of 500 mm/min under the same environmental conditions. The ASTM D638 method has been used in several studies for characterizing PCL-based systems [30,31].

For each sample, at least six separate specimens were tested, and the resulting stress–strain data are reported as mean values together with their corresponding standard deviations. The stress was calculated using the measured cross-sectional area, which normalizes thickness in the stress calculations. Young’s modulus was calculated in accordance with the ASTM standard used for that polymer matrix. Within each polymer matrix, all compositions were characterized under identical testing conditions (PLA series: ASTM D882; PCL series: ASTM D638), ensuring consistent comparison of the effect of PoPE content. Direct quantitative comparison of absolute tensile properties between PLA and PCL should therefore be considered exploratory due to the different specimen geometries and testing standards.

##### Thermogravimetric Analysis (TGA)

For thermogravimetric analysis (TGA) NETZSCH TG 309 Classic (NETZSCH-Gerätebau GmbH, Selb, Germany) was used. Rectangular pieces were cut from films, and 5–7 mg of each sample were placed in open ceramic crucibles. The measurements were performed in a temperature range from 30 to 700 °C with a heating rate of 10 K/min under a constant nitrogen flow of 250 mL/min. For each sample, at least two runs were recorded to check reproducibility.

##### Differential Scanning Calorimetry (DSC)

Differential scanning calorimetry (DSC) measurements were conducted using a Mettler Toledo DSC 1 STAR System (Mettler-Toledo GmbH, Greifensee, Switzerland). The analysis involved two distinct thermal cycles performed under a constant nitrogen gas flow of 50 mL/min to maintain an inert atmosphere. Samples with a mass of 10 ± 1 mg were placed in 100 μL crucibles. The thermal program consisted of a heating cycle from 25 to 220 °C. The heating cycle was executed at a constant rate of 10 °C/min. The DSC-derived melting (T_m_), cold crystallization (T_cc_), and T_g_ temperatures have an accuracy of ±2 °C. The characteristic transition temperatures were determined and analyzed using the associated STARe software (Version 8).

##### X-Ray Diffraction (XRD)

The crystalline structures of the samples were investigated using a Bruker D8 Advance X-ray diffractometer (Karlsruhe, Germany) equipped with Cu Kɑ radiation (*λ* = 1.5418 Å). The generator was operated at a voltage of 40 kV and a current of 40 mA. XRD patterns were recorded at ambient temperature in the 2*θ* range of 10° to 90°. The measurements were conducted in “Locked Coupled” scan mode with a step size (increment) of 0.0198° and a constant scan rate of 2°/min.

##### Scanning Electron Microscope (SEM)

The surface morphology of PLA and PCL films containing PoPE was analyzed using a Thermo Scientific Phenom XL scanning electron microscope (SEM) (Waltham, MA, USA). Samples were coated with gold and examined at a magnification of 1500× with an accelerating voltage of 5 kV.

##### Statistical Analysis

The results are expressed as the mean ± standard deviation by descriptive statistical methods using IBM SPSS Statistics 26 with Analysis of Variance (ANOVA). Tukey test was used to analyze multiple comparisons between samples at a confidence level of 95% (*p* < 0.05). All analyses were performed at least in duplicate.

## 3. Results and Discussion

### 3.1. Characterization Results of PoPE

#### 3.1.1. Extraction Efficiency

The extraction yields obtained with different solvents and methods are presented in Table 2, and the results varied between 7.35% and 29.80%. The most efficient extraction was achieved with a 30 min ultrasound-assisted extraction using water as the solvent, and the extraction yield was found to be 29.80% (W_US_30 min), followed by 24 h of maceration with 60% ethanol. The least efficient extraction was achieved with a 1 h maceration using ethanol, and the extraction yield was found to be 7.35% (E_1 h). In a study, Malviya et al. [32] extracted pomegranate peel powder using various extraction solvents such as ethanol, methanol, water, and a mixture of ethanol–water at different concentrations. Accordingly, the most efficient extraction method was stated as 50% ethanol with 24 h of maceration, and the yield was found to be 16.28%. The results from the literature and the current study agree with each other. In a study conducted by Kennas and Amellal-Chibane [33], PoPE was obtained with ethanol, methanol, water, acetone, and a mixture of water/methanol (50:50, *v*/*v*), and the highest yield was obtained with a mixture of water/methanol, followed by ethanol and methanol.

#### 3.1.2. Total Phenolic Content

The total phenolic content (TPC) results of the PoPE prepared using different methods are presented in Table 2 and range from 56.04 to 295.34 GAE/g. The highest TPC was obtained when ethanol extraction was performed for 24 h (E_24 h) and was calculated as 295.34 ± 9.31 GAE/g (*p* < 0.05). The lowest TPC was obtained when ethanol–acid–ultrasound extraction was performed for 10 min (EA_US_10 min) and was calculated as 56.04 ± 0.45 GAE/g. In a study by Mashkor [34], the highest total phenolic content of PoPE was obtained when acetone extraction was performed and was reported as 168.26 mg GAE/g, showing significantly lower yield than the 24 h ethanol extraction method chosen in this study. In the same study, the lowest yield was obtained by the extraction using water as the solvent, which was determined to be 84.15 mg GAE/g. In another study [33], the highest phenolic content was obtained with ethanol and a water/methanol mixture.

#### 3.1.3. Antioxidant Activity

The antioxidant activity of PoPE using different methods, measured using the DPPH and ABTS methods, is given in Table 2. The concentration range measured using the DPPH method is 17.70 ± 1.31–46.49 ± 1.72 mM Trolox/g, with the highest concentration shown in E_24 h, while the lowest is shown in W_US_10 min (*p* < 0.05). The concentration range measured using the ABTS method is 16.73 ± 1.36–55.68 ± 1.19 mM Trolox/g. The highest concentration was observed in E_24 h, and the lowest in W_24 h. In a study by Kennas and Amellal-Chibane [33] on the DPPH and ABTS methods, ethanol showed the highest antioxidant capacity in the DPPH method (76.75 ± 2.59 μg/mL). For ABTS, ethanol also showed the highest result (78.92 ± 1.13%). In another study by [35], methanol was found to have the highest antioxidant capacity, followed by ethanol in PoPE obtained by ultrasound-assisted extraction. The results of these studies correlate with those of the current study.

According to the total phenolic and antioxidant activity results as well as extraction efficiency, 24 h maceration with 60% ethanol (E_24 h) was selected as the optimal extraction method. Therefore, a 24 h maceration of PoPE with 60% ethanol was used for film preparation.

### 3.2. Characterization of PLA and PCL Films

#### 3.2.1. FTIR

The FTIR spectra of both the PLA and PCL films exhibit the characteristic absorption bands of the respective polyesters, confirming that the polymer backbones are preserved after incorporation of PoPE.

**Figure 1:**
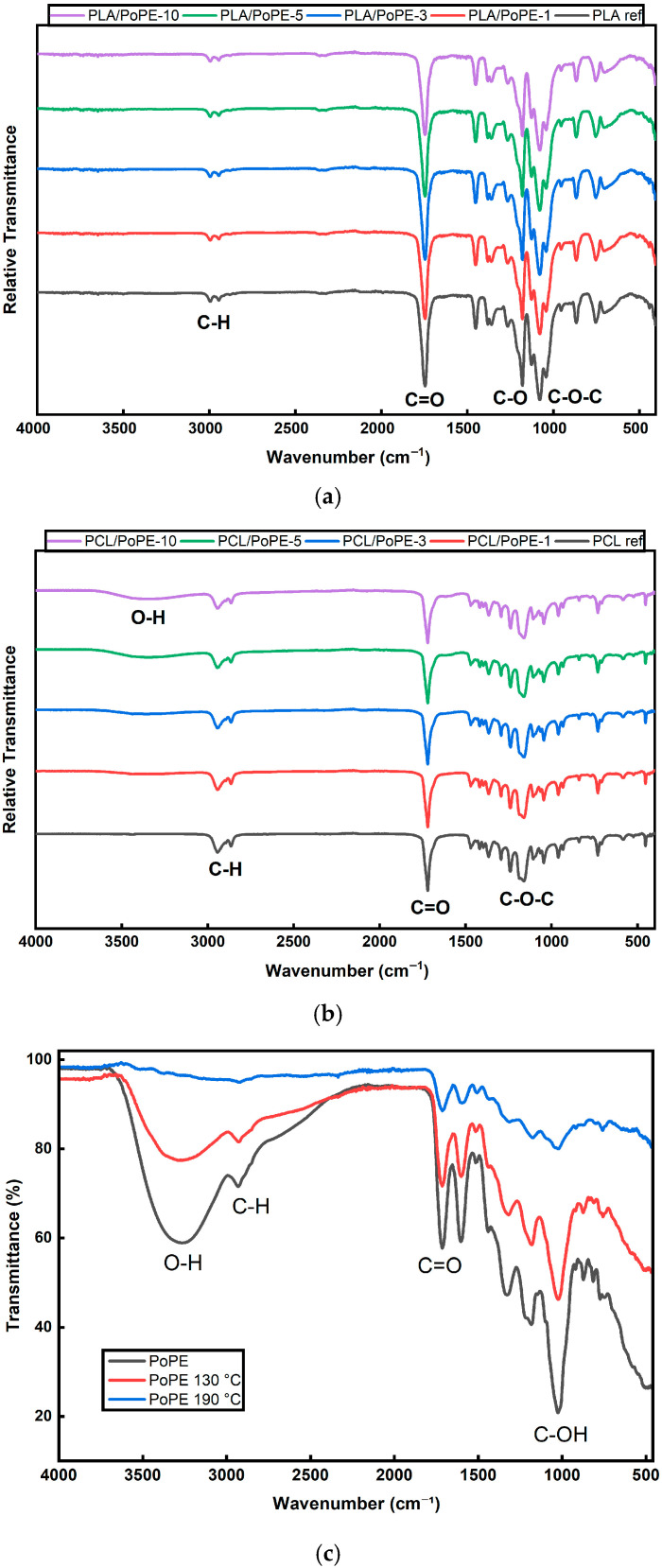
FTIR results of PoPE-containing PLA films (**a**), PoPE-containing PCL films (**b**), PoPE before heating and after thermal conditioning at 130 and 190 °C (**c**).

Figure 1a shows that in the PLA films, the symmetric and asymmetric stretching of aliphatic C–H bonds appear in the 3000–2940 cm^−1^ region, and the crucial ester C=O group gives a very strong, sharp peak at ~1750 cm^−1^ [36,37]. Prominent absorption bands corresponding to C–O stretching and C–O–C asymmetric vibrations are visible in the 1300–1000 cm^−1^ region, indicating ester links in the polymer [36]. PoPE was added at 1, 3, 5, and 10 wt.%, yet the FTIR analyses did not show detectable spectral changes with increasing PoPE content. The locations of the primary PLA peaks and their relative intensities remained nearly constant throughout all composite films, suggesting that the components most likely exist as a physical blend without significant chemical interactions between PoPE and the PLA matrix.

Figure 1b shows that in the PCL films, the intense band at ~1720 cm^−1^ corresponds to the ester carbonyl (C=O), while peaks near ~2940 and ~2865 cm^−1^ arise from the asymmetric and symmetric stretching of aliphatic –CH_2_ groups along the caprolactone backbone. Below 1300 cm^−1^, the strong bands at ~1295, ~1240, ~1190–1160, ~1100, and ~1040 cm^−1^ are associated with C–O and C–C stretching and C–O–C vibrations of the ester linkage in PCL, in agreement with the literature [38]. The fact that these bands remain essentially unchanged in position and shape across all PoPE loadings indicates that blending with PoPE did not cause chain scission or other chemical degradation of the PCL matrix during processing. By contrast, the main spectral differences between PCL ref and the PoPE-containing films appear in the high-wavenumber region (≈3600–3000 cm^−1^), where a broad O–H envelope grows with PoPE content. This can be attributed to phenolic hydroxyls, residual polysaccharides, and other bioactive components in pomegranate peels, indicating the successful incorporation of PoPE. The broadened O–H band is consistent with hydrogen bonding between phenolic –OH groups and PCL carbonyls.

PLA and PCL films contained the same PoPE, yet the FTIR spectra in Figure 1a,b showed small differences, particularly in the broad O–H stretching region. To verify whether these differences were driven by processing temperature rather than composition, PoPE was thermally conditioned at 190 °C and 130 °C and then analyzed by FTIR. According to result, PoPE has a broad band in the ~3000–3500 cm^−1^ region, corresponding to the stretching vibration bond of the O–H groups of polyphenols, mainly gallic acid, as well as bands at ~1600 and ~1200 cm^−1^. There is a small bond at ~2950 cm^−1^ assigned to the stretching of aliphatic C–H groups. The band at ~1700 cm^−1^ can be attributed to carbonyl groups (C=O), while the bands at ~1440 and ~1330 represent aromatic rings. The intense band at ~1000 cm^−1^ corresponds to the stretching of alcoholic groups (C–OH). The bands of functional groups of PoPE were consistent with another study [36]. When PoPE was processed at 130 °C and 190 °C, the intensity of the bands decreased due to the thermal degradation of the functional groups of phenolic compounds. Also, thermal processes promote Maillard reactions, the reaction of phenolic compounds with sugar fragments, resulting in the transformation of hydroxyl-bearing compounds [39]. Especially at 190 °C, significant degradation of the O–H groups of polyphenols can be seen in Figure 1c. This explains why the addition of PoPE does not cause any changes in the PLA bands, as PLA films were produced at 190 °C. Similarly, processing at 130 °C caused a decrease in the bands, especially in O–H groups, but they still existed in PoPE, which is consistent with the formation of a broadened O–H band in PoPE-containing PCL films. In a study conducted by Gurbanov et al. [40], PoPE processed at 105 °C for 30 min led to a significant change in the chemical composition of PoPE, which was mainly caused by the denaturation of organic compounds in PoPE.

Overall, FTIR confirms that PoPE incorporation does not alter the fundamental chemical structures of either matrix. The diagnostic ester carbonyls and fingerprint bands remain at their typical positions for both PCL and PLA. Additive-related changes are limited to the broad O–H region, which is more pronounced in the PCL films than in PLA, consistent with non-covalent interactions in PCL and largely physical incorporation in PLA, without the formation of new covalent bonds in either system.

#### 3.2.2. UV-Vis Spectroscopy

**Figure 2:**
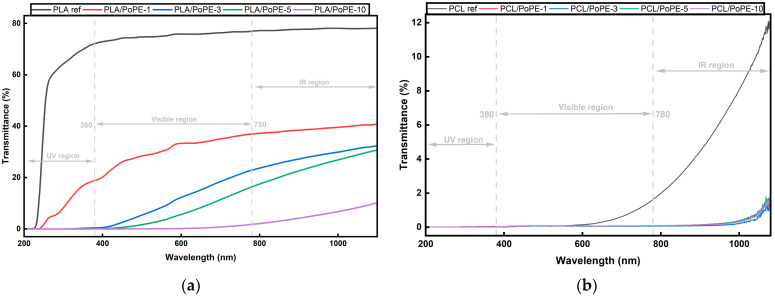
UV-Vis spectra result of the PLA films (**a**), and PCL films (**b**).

Both PLA and PCL films show that PoPE lowers optical transmittance across the spectrum, but the starting points of the two matrices differ, as is evident in Figure 2. PLA ref exhibits high visible transmittance and limited UV blocking, consistent with the intrinsic clarity of PLA films. As PoPE is added, transmittance decreases progressively over the whole spectrum. In the UV spectrum, all PoPE-containing PLA films except 1 wt.% approach ~0% transmittance, evidencing effective UV shielding, while in the visible region, PLA’s high transparency drops with increasing PoPE.

By contrast, PCL behaves as a strong light barrier from the outset: in the UV region, all PCL films, including the PCL ref, absorb essentially all incident light. In the visible region, transmittance remains very low at all PoPE levels, reflecting added absorption by the phenolics together with increased light scattering from pigment/filler domains. In the IR region, PCL ref rises to ~12% transmittance, whereas PoPE reduces this to ~1–2%, further enhancing the overall light-barrier performance [38,41].

These spectral changes are mainly driven by PoPE polyphenols (ellagitannins, flavonoids, anthocyanin-type pigments), which strongly absorb in the UV and partly in the visible region [23,42].

The polymer morphology explains the significantly lower transmittance of the PCL reference sample compared to the PLA reference sample. Luminy^®^ LX175 PLA is an amorphous polymer, while PCL has a semi-crystalline structure. Therefore, the low transmittance of PCL is primarily attributed to its semi-crystalline microstructure, while thickness differences may be a secondary contributing factor.

Taken together, UV–Vis indicates that PoPE provides a tunable, clean-label route to warm coloration and effective UV protection, which are both desirable in active food packaging, while reducing transparency as an expected consequence of phenolic absorption and micro-scale scattering [43,44]. In practice, ~3–5 wt.% PoPE in PLA is sufficient to achieve near-total UV absorption for light-sensitive products, whereas PCL already delivers very high UV shielding and requires only very low PoPE loading to reach a comparable barrier level.

#### 3.2.3. Color Analysis

The color data in Table 3 shows a clear, concentration-dependent shift with PoPE in both matrices. As PoPE increases, lightness (L*) decreases, yellowness (b*) rises, chroma (C*) grows, and the hue angle moves toward yellow/orange. These trends are consistent with the visible absorption of PoPE polyphenols (ellagitannins, flavonoids, anthocyanin-type pigments) and align with the UV–Vis observations.

For PLA ref, L* is 97.52 and it falls to 73.93 at 10 wt.%, b* increases from 0.26 to 34.17, and a* remains near zero up to 3 wt.% before increasing to 7.61 at 10 wt.%. Accordingly, C* rises from 0.26 to 35.00, and the hue shifts from ~86° toward ~77° (yellow-orange), reflecting a transition from almost transparent to an amber-brown tint.

For PCL, the reference is very light, slightly reddish, and weakly yellow which is consistent with the nearly white/cream look usually reported for PCL [38]. With the addition of PoPE, the dominant change is the rise in b*. The b* value is 4.17 for the PCL ref, and it reaches 15.57 at 10 wt.%. The hue angle shifts from 69.28° toward ~82°, approaching the “pure yellow” region, indicating a more vivid yellow rather than a neutral tone.

The ΔE* quantifies the visual shift in the films. In PLA films, ΔE* is 5.05 at 1 wt.%, 13.35 at 3 wt.%, 28.56 at 5 wt.%, reaching 42.00 at 10 wt.% compared to PLA ref. For the PCL films, it lies between 4.02 and 15.79 across PoPE levels. Thus, even 1 wt.% is visibly different from the reference in both matrices, and higher loadings yield a strong yellow-amber appearance. The color change in the films is higher than 3, which means that the color change is visually noticeable to the human eye [26]. Larger standard deviations at higher PoPE contents are consistent with dispersion heterogeneity of plant-extract domains affecting optical results [45].

Overall, PoPE enables tunable, clean-label coloration without synthetic dyes while simultaneously supporting UV protection evident in UV-Vis results [45,46]. This reduces concerns about dye migration and aligns with bio-based packaging goals, offering shade control via PoPE content alongside functional light-barrier benefits [47]. The strong coloration and reduced transparency at high PoPE loadings may restrict use in applications requiring clear packaging.

#### 3.2.4. Tensile Test

**Figure 3:**
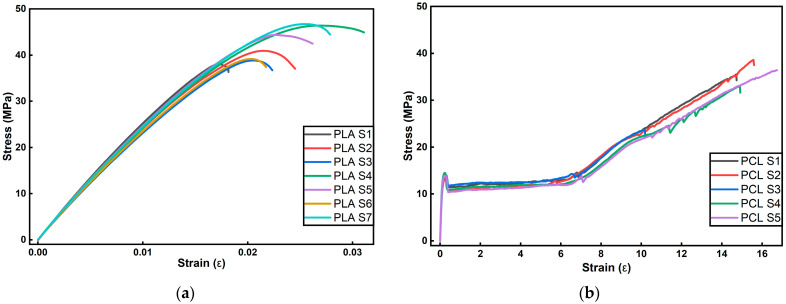
Stress–strain graphs of PLA (**a**) and PCL (**b**) films.

Before interpreting the effect of PoPE, it is important to note that neat PLA and neat PCL exhibit intrinsically different deformation mechanisms, especially in elongation. Figure 3a,b show the stress–strain curves of repeated measurements of PLA and PCL reference samples, respectively, to demonstrate the mechanical behavior of neat PLA and PCL films at room temperature. PLA typically behaves as a stiff, high-strength but brittle polymer at room temperature (Tg ≈ 55 °C) and is often reported to fail at low strains, which matches the PLA curves in Figure 3a that reach ~40–47 MPa but fracture around ~2–3% strain. In contrast, PCL is rubbery at room temperature (Tg ≈ −60 °C) and can sustain very large plastic deformation, consistent with the extended yielding and strain-hardening regime seen in the PCL curves in Figure 3b.

As is evident in Table 4, across both matrices, PoPE modifies the stress–strain response in matrix-dependent ways. The PLA films exhibit a strength/ductility maximum at low to medium loadings, the stress at break increases from 42.11 MPa on PLA ref to a peak of 50.35 MPa at 5 wt.%, while the strain at break reaches its maximum value of 0.07 at 3 wt.%. Within this 1–5 wt.% range, PoPE behaves as a mild plasticizer, enhancing chain mobility and interfacial stress transfer, in line with the previously reported PLA films containing PoPE or other vegetal polyphenol extracts [23,48]. A similar effect was also observed in previous studies, where mechanical properties deteriorate beyond an optimal filler content due to particle clustering and local stress concentrations [49].

In contrast, the PCL matrix responds differently to PoPE addition, as its stress at break remains relatively stable across the series, peaking slightly at 1 wt.% to 38.59 MPa and remaining near the reference value of 35.12 MPa even at higher loadings. The ductility of the PCL films remains essentially unchanged with strain at break values between 15.5 and 16.9, confirming that the addition of PoPE does not cause embrittlement. This retention of flexibility aligns with studies on PCL modified with plant extracts, where mechanical robustness is achieved without sacrificing the elasticity required for applications such as wound dressings or packaging components [24,50].

Although changes were observed in the Young’s modulus values, the magnitude of these variations was not very pronounced. As expected, the PLA films exhibited relatively high modulus values, consistent with the inherently stiff and brittle nature of PLA. In contrast, the PCL films showed lower modulus values, which can be attributed in part to the fact that they were produced without any prior stretching or drawing step. As a result, the PCL samples underwent plastic deformation under relatively low loads and fractured only after large elongations. It is well known that applying a stretching step during processing can increase molecular orientation in PCL, which may in turn improve its modulus values.

Overall, PoPE creates distinct mechanical performance windows in the two polymers. In PLA, 3–5 wt.% PoPE appears to be the most favorable range for balancing higher tensile strength and improved elongation. For PCL films, intrinsic strength and high flexibility are maintained across 1–10 wt.%. For packaging, these windows provide practical tunability. If higher load bearing is desired, PLA + PoPE (3–5 wt.%), and if flexible handling with relatively lower load bearing is prioritized, PCL + PoPE (1–10 wt.%) can be chosen.

#### 3.2.5. TGA

TGA was performed to investigate the impact of PoPE incorporation on the thermal stability of PLA- and PCL-based films, which is crucial for melt-processing and their application as bio-based packaging polyesters. TGA is routinely used to characterize the decomposition behavior of PLA and PCL and to compare neat polymers with active, plant-extract-containing formulations intended for food packaging applications.

**Figure 4:**
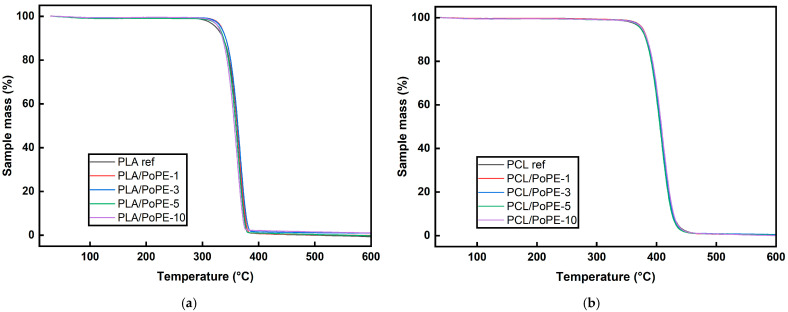
TGA graphs of PLA (**a**) and PCL (**b**) films.

For the PLA-based systems, all curves in Figure 4a show a very similar thermal behavior. The PLA reference and the PoPE-containing films remain essentially stable up to around 300 °C, with almost no mass loss in the processing and service temperature range relevant for packaging. A single, sharp degradation step is then observed, with the main weight loss occurring in a narrow interval at high temperature and completed by ~400 °C, consistent with the one-step decomposition typically reported for PLA films [51,52]. The onset and maximum degradation temperatures of the PoPE-containing PLA films are practically unchanged with respect to PLA ref, indicating that incorporation of up to 10 wt.% pomegranate-peel extract does not compromise the thermal stability of the matrix. This behavior is in line with other PLA systems containing vegetal polyphenol extracts, where the main decomposition step remains at similar temperatures and only modest shifts in degradation temperature are observed as the additive loading is increased [23,48]. Only a slight increase in the final residue is visible at the highest PoPE contents, which can be attributed to the small amount of non-volatile components in the extract, as also stated in recent studies for other polyphenol-modified PLA films [48].

For the PCL-based films, the TGA curves in Figure 4b are almost identical for all compositions. Almost no weight loss is observed until the main degradation begins, which occurs at ~360 °C and is completed at ~460 °C. Such single-step decomposition with high degradation temperatures is characteristic of PCL and PCL-based active films [7,53]. The presence of PoPE of up to 10 wt.% does not significantly alter the onset or the rate of decomposition, and the final residue remains very low, with only a marginal increase compared to the PCL ref. Similar observations have been reported for PCL films containing plant- or fruit-derived extracts, where the antioxidant additives only slightly affect the degradation temperatures while preserving the overall thermal profile of PCL [13]. These results demonstrate that PoPE is thermally compatible with the PCL matrix and does not cause early degradation.

Overall, the TGA results indicate that PoPE can be incorporated into PLA and PCL films at least up to 10 wt.% without any significant effects on their thermal stability. Both series remain stable throughout typical processing temperatures and degrade in a single step at high temperatures, with only minor increases in residue associated with the non-volatile fraction of the extract. This thermal robustness, combined with the known antioxidant and antimicrobial potential of PoP-derived additives in polymer films, supports the suitability of PoPE-containing PLA and PCL as promising candidates for thermally demanding active food-packaging applications [54,55].

#### 3.2.6. DSC

Table 5 summarizes the DSC-derived transition temperatures and crystallinity of PLA and PCL films. For PLA films, the T_g_ remains essentially unchanged, which is between 61.59 and 62.71 °C, indicating that PoPE does not markedly plasticize the PLA amorphous phase under the present processing conditions. T_g_ range is consistent with commonly reported PLA values [56]. All PLA formulations exhibit cold crystallization (T_cc_) between 120 and 124.14 °C. This confirms that films are very amorphous after processing. This amorphous nature, coupled with the high molecular weight of the polymer, is a key factor in the production of these highly transparent films, which are essential for functional food packaging applications [57]. The minor shifts in T_cc_ upon PoPE addition suggest that the extract does not act as a strong heterogeneous nucleating agent for PLA crystallization. In parallel, the melting temperature (T_m_) shows small changes, which occur between 149.17 and 151.67 °C. This means that PoPE addition has no meaningful impact on the characteristic temperature values of the PLA. The calculated crystallinity of the PLA films is extremely low, and it is around 3–5%. The findings in the literature for crystallinity of PLA LX175 are also very similar [58,59]. These results indicate that these processing conditions did not allow PLA films to crystallize.

In contrast, the PCL films displayed the expected semicrystalline behavior and showed a minor increase in thermal stability of the crystalline phase with PoPE loading. The melting temperature increased from 62.5 °C for PCL ref to 66.83 °C for PCL/PoPE-10, accompanied by a monotonic increase in crystallinity from 56.60% to 62.22%. The T_g_ temperature of PCL was around −60 °C, which is way beyond the DSC analysis temperature range used in this study. Also, PCL films did not show any T_cc_.

Overall, the DSC results indicate a clear matrix-dependent effect of PoPE. The PLA films remained essentially amorphous after processing, as evidenced by a persistent cold-crystallization event and near-zero net crystallinity, with only minor shifts in T_g_ and a slight decrease in T_m_ upon PoPE addition. In contrast, PoPE systematically increased the melting temperature and crystallinity of PCL, suggesting enhanced crystal formation and/or improved crystal stability at higher extract loadings. These findings demonstrate that PoPE does not promote net crystalline development in PLA under the applied conditions, whereas it acts as an effective crystallization-promoting additive in PCL.

#### 3.2.7. XRD

**Figure 5:**
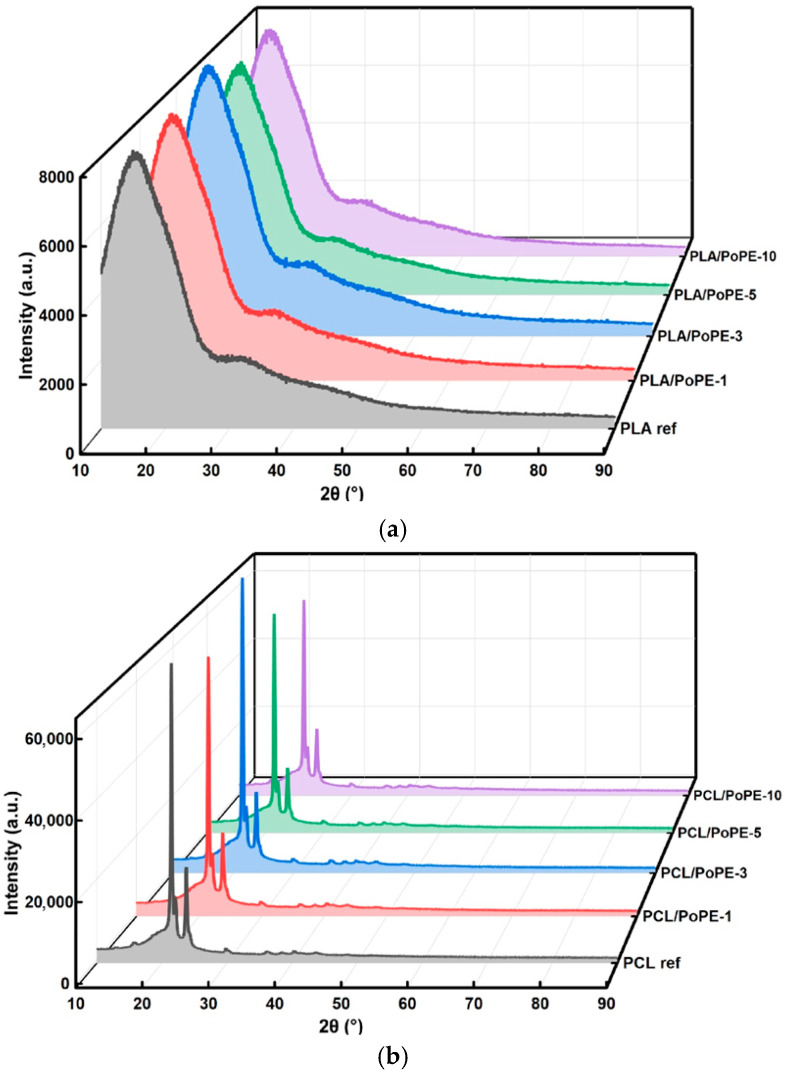
XRD graphs of PLA (**a**) and PCL (**b**) films.

The XRD patterns in Figure 5 provide structural support for the DSC results and show the strong contrast between PLA and PCL crystallization. The PLA diffractograms display a dominant broad amorphous halo around the 2θ ≈ 15–25° region with no clear sharp reflections. Well-crystallized PLA typically shows characteristic peaks around 2θ ≈ 14.8°, 16.7°, 19.0–19.1°, and 22.2–22.3°, which are assigned to common α-form planes, whereas rapidly cooled or poorly crystallized PLA often appears largely amorphous by XRD [56,60]. The absence of these α-form reflections in PLA films is consistent with the DSC results, which are near-zero crystallinity and the presence of cold crystallization at ~120–124 °C. In addition, PoPE does not introduce new crystalline reflections in the PLA patterns, suggesting that either its crystalline contribution is below detection relative to the polymer background or the PLA lattice is not transformed into a different crystalline polymorph by PoPE addition. This outcome aligns with prior PLA active-film studies incorporating PoPE, in which property changes are often attributed to interfacial interactions and dispersion rather than to the formation of new polymer crystal phases [23].

For PCL, the diffractograms show intense, sharp reflections characteristic of orthorhombic PCL, most notably near 2θ ≈ 21.4°, 22.1°, and 23.7°, corresponding to the (110), (111), and (200) planes, respectively [38,61]. Across the PCL/PoPE series, these crystalline peaks remain at essentially the same 2θ positions, indicating that the PCL crystal structure is retained. Minor additional weak reflections appearing in the PCL/PoPE series could plausibly originate from low-level crystalline residues within the natural extract. However, because the main PCL reflections are unchanged in position, these features do not alter the interpretation that the polymer maintains its orthorhombic crystalline identity.

Overall, XRD indicates that PoPE does not change the fundamental crystal structure of either polymer. PLA remains largely amorphous under the applied processing history, while PCL retains its orthorhombic crystalline phase. The main structural impact of PoPE is expected to result primarily as changes in intensity/broadening effects rather than new polymer crystalline polymorphs.

#### 3.2.8. SEM

**Figure 6:**
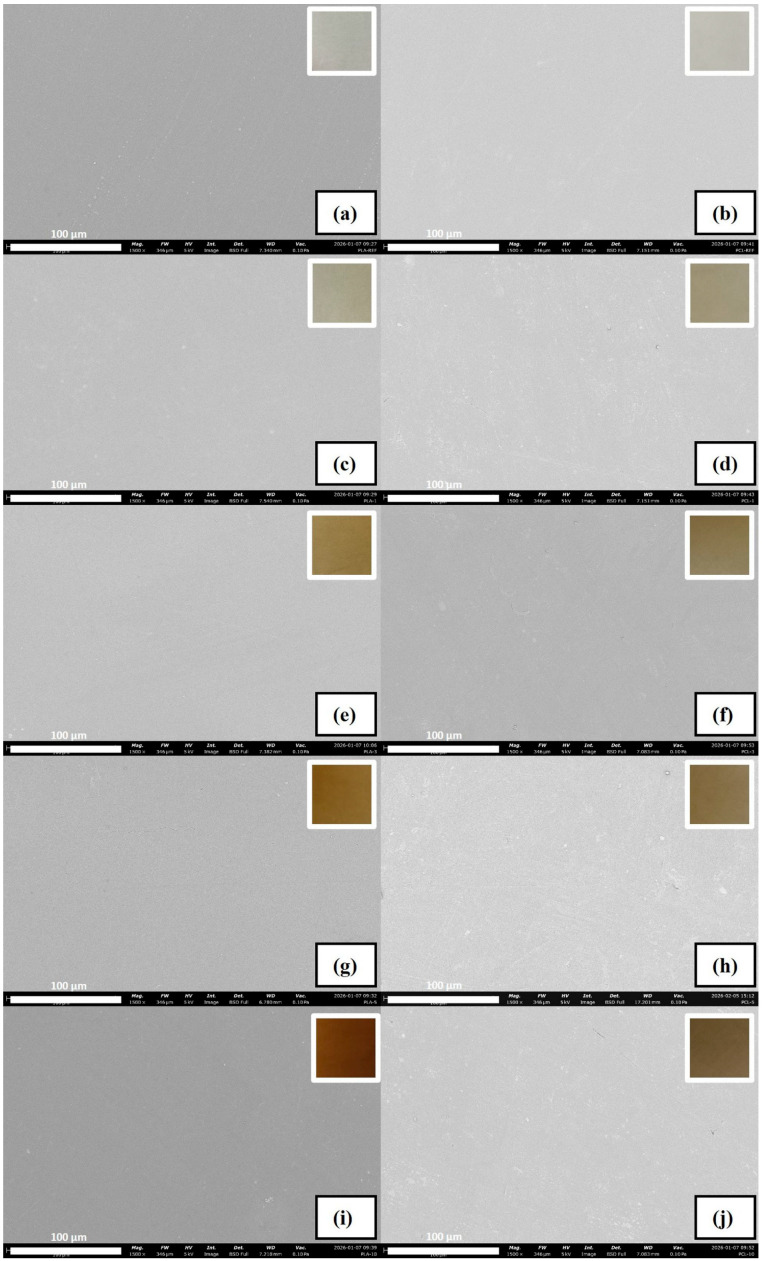
SEM images of films (PLA ref: (**a**); PLA/PoPE-1: (**c**); PLA/PoPE-3: (**e**); PLA/PoPE-5: (**g**); PLA/PoPE-10: (**i**); PCL ref: (**b**); PCL/PoPE-1: (**d**); PCL/PoPE-3: (**f**); PCL/PoPE-5: (**h**); and PCL/PoPE-10: (**j**) at magnification of 1500×).

The morphology of PLA and PCL films was examined using SEM images shown in Figure 6a–j. The photographs of the corresponding films were captured in the same conditions with a white background and included as insets in the upper-right corner of each image.

SEM images revealed a consistent and robust structure without any agglomeration, pores or cracks, displaying homogeneous dispersion of PoPE in PLA and PCL films. Similarly, there was no non-homogeneity in the films when looked at with the naked eye. The homogeneous distribution observed by SEM can be attributed to the chemical structure of PoPE. PoPE contains punicalagins, ellagic acid, and hydrolysable tannins; these components exhibit amphiphilic properties due to the presence of both hydrophilic hydroxyl groups and hydrophobic aromatic domains [14,15,62,63,64]. Consequently, PoPE can participate in hydrogen bonding and hydrophobic interactions. Therefore, it is thought that these features may have facilitated its integration into hydrophobic polymer matrices such as PLA and PCL, thus enabling the formation of a homogeneous structure. In a study by Lyu et al. [38], PCL film incorporated with grape seed extract at 1, 3, and 5% (*w*/*w*) concentrations showed a similar smooth surface on SEM images, resulting in homogeneity in films.

## 4. Conclusions

This study demonstrates that PoPE serves as a multi-functional bio-additive that relatively enhances the performance of PLA and PCL films without compromising their fundamental structural integrity. Morphological and structural characterizations via SEM reveal a highly homogeneous dispersion of the extract across loading levels up to 10 wt.%, with no evidence of agglomeration, pores, or cracks. FTIR analysis further supports this stability, indicating that the polymer backbones remain unaltered.

Thermal stability assessments via TGA prove that these PLA and PCL films are robust enough for demanding industrial processing, maintaining stability throughout typical temperature windows. However, the influence of PoPE on crystallization is matrix-dependent; while PLA remains essentially amorphous with near-zero net crystallinity, PoPE acts as a crystallization-promoting agent in PCL, slightly increasing its melting temperature and crystalline development.

From a functional perspective, PoPE offers a tunable route to functionalize biodegradable PLA and PCL films by introducing a natural, bio-based additive that imparts active packaging attributes. It provides exceptional UV shielding and imparts a natural warm coloration that eliminates the need for synthetic dyes. Mechanically, PoPE enhances the toughness of PLA at 3–5 wt.% and maintains the high ductility of PCL across all loading levels, offering practical tunability for different load-bearing requirements.

The resulting opacity increase caused by PoPE is application-dependent; it is highly beneficial for keeping light-sensitive foods (e.g., essential oils, dairy products, processed meat products, wine, dried fruits) fresh by improving UV/light protection, but it may limit use in packaging formats where high transparency and product visibility are required. Thus, PoPE loading should be chosen by balancing appearance and protective performance.

While this research establishes a comprehensive understanding of the processing–structure–property relationships of PoPE-incorporated films, further investigations are essential to fully validate their potential as promising active packaging candidates. Future work will focus on quantifying the antioxidant activity of films and controlled release kinetics, alongside systematic assessments of barrier performance, including oxygen transmission rates (OTR) and water vapor transmission rates (WVTR). Additionally, long-term aging, assessment of antimicrobial activity, and the impact on the quality characteristics of food during shelf life will be evaluated. Finally, comprehensive migration behavior and food-contact compliance testing will be conducted in relevant food-packaging models to ensure material safety and comprehensive functionality.

## Figures and Tables

**Table 1:** Names, additive ratios, and thickness of all samples.

Sample Name	Additive wt. (%)	Thickness (mm)
PLA ref	0	0.15 ± 0.02
PLA/PoPE-1	1	0.19 ± 0.03
PLA/PoPE-3	3	0.22 ± 0.03
PLA/PoPE-5	5	0.20 ± 0.02
PLA/PoPE-10	10	0.17 ± 0.02
PCL ref	0	0.38 ± 0.04
PCL/PoPE-1	1	0.34 ± 0.05
PCL/PoPE-3	3	0.31 ± 0.05
PCL/PoPE-5	5	0.25 ± 0.01
PCL/PoPE-10	10	0.29 ± 0.04

**Table 2:** Results of extraction yield, total phenolic content (TPC), and antioxidant activity (DPPH and ABTS) of PoPE obtained by different extraction methods.

Sample	Extraction Yield (%)	TPC (mg GAE/g)	DPPH (mM TE/g)	ABTS (mM TE/g)
E_1 h	7.35	218.92 ± 8.76 ^c^	40.97 ± 1.55 ^bcd^	44.37 ± 3.96 ^bc^
E_24 h	23.01	295.34 ± 9.31 ^a^	46.49 ± 1.72 ^a^	55.68 ± 1.19 ^a^
M_1 h	12.46	206.01 ± 3.62 ^c^	39.48 ± 0.01 ^cd^	47.49 ± 1.16 ^b^
M_24 h	26.18	268.98 ± 11.27 ^b^	44.76 ± 2.35 ^ab^	46.60 ± 4.06 ^b^
W_ 1 h	15.44	69.78 ± 2.58 ^e^	24.64 ± 1.49 ^e^	26.58 ± 1.01 ^def^
W_24 h	25.70	109.98 ± 1.09 ^d^	23.20 ± 1.48 ^ef^	16.73 ± 1.36 ^g^
W_US_10 min	24.69	269.92 ± 13.24 ^e^	17.70 ± 1.31 ^g^	32.04 ± 4.45 ^d^
W_US_20 min	21.59	252.05 ± 8.71 ^e^	19.46 ± 0.01 ^fg^	24.58 ± 0.14 ^ef^
W_US_30 min	29.80	269.46 ± 8.84 ^e^	18.13 ± 0.41 ^g^	22.55 ± 2.15 ^fg^
EA_US_10 min	28.84	56.04 ± 0.45 ^b^	42.20 ± 1.56 ^abc^	44.09 ± 1.12 ^bc^
EA_US_20 min	10.98	66.72 ± 1.52 ^b^	36.59 ± 1.33 ^d^	31.15 ± 2.01 ^de^

Extraction solvents: E: ethanol, M: methanol, W: water, EA: ethanol–acid; US: ultrasound-assisted extraction. Extraction times: 1 h: 1 h; 24 h: 24 h; 10 min: 10 min; 20 min: 20 min; 30 min: 30 min. Small letters (a–g) represent significant differences for each sample in the same column (*p* < 0.05).

**Table 3:** Color analysis results of the PLA and PCL films.

Sample	L*	a*	b*	C*	Hue°	ΔE*	Color
PLA ref	97.52 ± 0.03 ^a^	0.02 ± 0.01 ^c^	0.26 ± 0.02 ^e^	0.26	86.28	-	
PLA/PoPE-1	95.24 ± 0.16 ^ab^	0.05 ± 0.01 ^c^	4.76 ± 0.38 ^d^	4.76	89.40	5.05	
PLA/PoPE-3	91.92 ± 0.22 ^b^	0.48 ± 0.06 ^c^	12.37 ± 0.66 ^c^	12.38	87.78	13.35	
PLA/PoPE-5	84.04 ± 2.18 ^c^	3.03 ± 0.79 ^b^	25.26 ± 3.21 ^b^	25.44	83.17	28.56	
PLA/PoPE-10	73.93 ± 2.07 ^d^	7.61 ± 0.71 ^a^	34.17 ± 2.02 ^a^	35.00	77.45	42.00	
PCL ref	94.62 ± 0.50 ^b^	1.58 ± 0.04 ^b^	4.17 ± 0.07 ^e^	4.46	69.26	-	
PCL/PoPE-1	97.09 ± 0.28 ^a^	0.90 ± 0.02 ^cd^	7.27 ± 0.16 ^d^	7.33	82.97	4.02	
PCL/PoPE-3	96.55 ± 0.47 ^ab^	0.86 ± 0.04 ^d^	9.07 ± 0.43 ^c^	9.11	84.58	5.31	
PCL/PoPE-5	94.88 ± 0.67 ^b^	1.12 ± 0.08 ^c^	10.65 ± 0.59 ^b^	10.71	84.00	6.50	
PCL/PoPE-10	83.71 ± 1.30 ^c^	2.25 ± 0.17 ^a^	15.57 ± 0.66 ^a^	15.74	81.78	15.79	

Small letters (a–e) represent that they differ significantly for each polymer matrix in the same column (*p* < 0.05).

**Table 4:** Tensile test results of the PLA and PCL films.

Sample	Stress at Break (MPa)	Strain at Break (ε)	Young’s Modulus (MPa)
PLA ref	42.11 ± 3.65 ^b^	0.03 ± 0.01 ^c^	2352.39 ± 135.16 ^ab^
PLA/PoPE-1	48.00 ± 2.31 ^a^	0.04 ± 0.01 ^bc^	2274.40 ± 185.45 ^ab^
PLA/PoPE-3	49.48 ± 1.61 ^a^	0.07 ± 0.02 ^a^	2107.42 ± 127.91 ^b^
PLA/PoPE-5	50.35 ± 1.95 ^a^	0.05 ± 0.01 ^ab^	2181.88 ± 209.09 ^b^
PLA/PoPE-10	42.45 ± 1.97 ^b^	0.03 ± 0.01 ^bc^	2531.97 ± 150.00 ^a^
PCL ref	35.12 ± 1.51 ^c^	15.51 ± 1.08 ^b^	150.26 ± 11.49 ^c^
PCL/PoPE-1	38.59 ± 0.54 ^a^	16.91 ± 0.02 ^a^	188.75 ± 4.33 ^a^
PCL/PoPE-3	37.80 ± 0.90 ^abc^	16.91 ± 0.03 ^a^	193.28 ± 6.13 ^a^
PCL/PoPE-5	37.97 ± 1.77 ^ab^	16.60 ± 0.63 ^ab^	163.36 ± 7.93 ^b^
PCL/PoPE-10	35.75 ± 2.20 ^bc^	16.58 ± 0.86 ^ab^	194.26 ± 5.09 ^a^

Small letters (a–c) represent that they differ significantly for each polymer matrix in the same column (*p* < 0.05).

**Table 5:** DSC results of the PLA and PCL films.

Sample	T_g_ (°C)	T_cc_ (°C)	T_m_ (°C)	Crystallinity (%)
PLA ref	61.59	120.00	151.67	4.20
PLA/PoPE-1	62.71	124.00	149.33	3.70
PLA/PoPE-3	62.65	124.17	149.67	5.80
PLA/PoPE-5	62.51	120.00	149.17	3.50
PLA/PoPE-10	62.05	123.67	150.00	4.40
PCL ref	-	-	62.50	56.60
PCL/PoPE-1	-	-	64.50	57.04
PCL/PoPE-3	-	-	65.50	57.37
PCL/PoPE-5	-	-	63.33	59.64
PCL/PoPE-10	-	-	66.83	62.22

## Data Availability

The original contributions presented in this study are included in the article. Further inquiries can be directed to the corresponding author.
